# Supplementary material for: Lactoferrin combined with Coenzyme Q10 ameliorate sarcopenia in an aging mouse model induced by D-galactose
Source: PLoS One. 2025 Jun 26;20(6):e0325297. doi: 10.1371/journal.pone.0325297 (PMC12200871; doi:10.1371/journal.pone.0325297)
Supplement: S1 Table — (DOCX) [file pone.0325297.s001.docx]

Contents of Supplemental Materials

APPENDIX 1 primers 1

Appendix 1 - primers

Table S1. Primers designed for quantitative Real-Time PCR .

| Primer | Sequence(5' to 3') |
| --- | --- |
| β-actin Forward | TGAGCTGCGTTTTACACCCT |
| β-actin Reverse | GCCTTCACCGTTCCAGTTTT |
| Myod1 Forward | AACTGTCCTTTCGAAGCCGT |
| Myod1 Reverse | TTGGGGCTGGATCTAGGACA |
| Myog Forward | CAGCCCAGCGAGGGAATTTA |
| Myog Reverse | AGAAGCTCCTGAGTTTGCCC |
| Myf5 Forward | GCAGCAGAAGAAACGTGTGAC |
| Myf5 Reverse | CATGGGGATGACAGTAGCTGAG |
| Mef2c Forward | GCACCAACAAGCTGTTCCAG |
| Mef2c Reverse | CTGAATCGTCTGCATCGGGA |
| Myoz2 Forward | GGCATCTGGAAACCTGGATGA |
| Myoz2 Reverse | GGCCGGTATGCAAGACACTT |
| Myh2 Forward | CGAAGAGTAAGGCTGTCCCG |
| Myh2 Reverse | GCGCATGACCAAAGGTTTCA |
| Fgf9 Forward | TCCTGTCTGGCTCTTAGGCT |
| Fgf9 Reverse | ATACAGCTCCCCCTTCTCGT |
| *Icam1* Forward | TTCTCATGCCGCACAGAACT |
| *Icam1* Reverse | TCCTGGCCTCGGAGACATTA |
| Sirt3 Forward | GTCCGGGAGTGTTACAGGTG |
| Sirt3 Reverse | ACCATGACCACCACCCTACT |
